# Efficacy of Three Low-Intensity, Internet-Based Psychological Interventions for the Treatment of Depression in Primary Care: Randomized Controlled Trial

**DOI:** 10.2196/15845

**Published:** 2020-06-05

**Authors:** Margalida Gili, Adoración Castro, Azucena García-Palacios, Javier Garcia-Campayo, Fermin Mayoral-Cleries, Cristina Botella, Miquel Roca, Alberto Barceló-Soler, María M Hurtado, MªTeresa Navarro, Amelia Villena, M Ángeles Pérez-Ara, Pau Riera-Serra, Rosa Mª Baños

**Affiliations:** 1 Institut Universitari d'Investigació en Ciències de la Salut University of Balearic Islands Palma de Mallorca Spain; 2 Institut d'Investigació Sanitaria Illes Balears Palma de Mallorca Spain; 3 Primary Care Prevention and Health Promotion Research Network RedIAPP Madrid Spain; 4 Department of Clinical and Basic Psychology and Biopsychology Faculty of Health Sciences Univeristy Jaume I Castellón Spain; 5 Biomedical Research Center Network (CIBER) Physiopathology Obesity and Nutrition (CIBERobn) Carlos III Health Institute Madrid Spain; 6 Departament of Psychiatry Hospital Miguel Servet University of Zaragoza Zaragoza Spain; 7 Mental Heath Unit Hospital Regional of Malaga Biomedicine Research Institute (IBIMA) Málaga Spain; 8 Aragón Institute for Health Research (IIS Aragon) Zaragoza Spain; 9 Department of Psychology and Sociology University of Zaragoza Zaragoza Spain; 10 Mental Health Unit of Pozoblaco Hospital Los Pedroches Córdoba Spain; 11 Department of Psychological, Personality, Evaluation and Treatment University of Valencia Valencia Spain

**Keywords:** depression, primary care, internet-based interventions, randomized controlled trial

## Abstract

**Background:**

Primary care is a major access point for the initial treatment of depression, but the management of these patients is far from optimal. The lack of time in primary care is one of the major difficulties for the delivery of evidence-based psychotherapy. During the last decade, research has focused on the development of brief psychotherapy and cost-effective internet-based interventions mostly based on cognitive behavioral therapy (CBT). Very little research has focused on alternative methods of treatment for depression using CBT. Thus, there is a need for research into other therapeutic approaches.

**Objective:**

This study aimed to assess the effectiveness of 3 low-intensity, internet-based psychological interventions (healthy lifestyle psychoeducational program [HLP], focused program on positive affect promotion [PAPP], and brief intervention based on mindfulness [MP]) compared with a control condition (improved treatment as usual [iTAU]).

**Methods:**

A multicenter, 4-arm, parallel randomized controlled trial was conducted between March 2015 and March 2016, with a follow-up of 12 months. In total, 221 adults with mild or moderate major depression were recruited in primary care settings from 3 Spanish regions. Patients were randomly distributed to iTAU (n=57), HLP (n=54), PAPP (n=56), and MP (n=54). All patients received iTAU from their general practitioners. The main outcome was the Spanish version of the Patient Health Questionnaire-9 (PHQ-9) from pretreatment (time 1) to posttreatment (time 2) and up to 6 (time 3) and 12 (time 4) months’ follow-up. Secondary outcomes included the visual analog scale of the EuroQol, the Short-Form Health Survey (SF-12), the Positive and Negative Affect Schedule (PANAS), and the Pemberton Happiness Index (PHI). We conducted regression models to estimate outcome differences along study stages.

**Results:**

A moderate decrease was detected in PHQ-9 scores from HLP (β=–3.05; *P*=.01) and MP (β=–3.00; *P*=.01) compared with iTAU at posttreatment. There were significant differences between all intervention groups and iTAU in physical SF-12 scores at 6 months after treatment. Regarding well-being, MP and PAPP reported better PHI results than iTAU at 6 months post treatment. PAPP intervention significantly decreased PANAS negative affect scores compared with iTAU 12 months after treatment.

**Conclusions:**

The low-intensity, internet-based psychological interventions (HLP and MP) for the treatment of depression in primary care are more effective than iTAU at posttreatment. Moreover, all low-intensity psychological interventions are also effective in improving medium- and long-term quality of life. PAPP is effective for improving health-related quality of life, negative affect, and well-being in patients with depression. Nevertheless, it is important to examine possible reasons that could be implicated for PAPP not being effective in reducing depressive symptomatology; in addition, more research is still needed to assess the cost-effectiveness analysis of these interventions.

**Trial Registration:**

ISRCTN Registry ISRCTN82388279; http://www.isrctn.com/ISRCTN82388279

**International Registered Report Identifier (IRRID):**

RR2-10.1186/s12888-015-0475-0

## Introduction

### Background

Depression represents a significant personal, economic, and societal burden [1-3]. Primary care remains a major access point for initial treatment of depression [4,5]. Previous studies reveal that more than 80% of patients with depression are being managed in general practice [6,7]. However, management of these patients is far from optimal, and it has been reported that only one-half of the patients receive adequate care, whether pharmacological or psychological [5]. Pence et al [4] estimated that only 47% of primary care patients with depression are clinically recognized, 24% receive treatment, and 9% receive adequate treatment.

Multiple and complex facilitators and barriers to treatment have been described [8,9], and access to evidence-based psychotherapy is one of these difficulties. Different factors such as professionals’ training, time needed, costs or work overload, professionals’ attitudes and organization, and geographical and logistic difficulties are some of the reasons that make the integration of psychotherapy in primary care difficult. New forms and models for delivering psychotherapy in primary care have been proposed to overcome these problems. The implementation of stepped and collaborative care models in primary care settings that provide time-limited psychotherapy have been found to improve the management of depression [10]. One of the most important difficulties for integrating psychotherapy into primary care is the lack of time and resources. Many empirically tested treatment protocols last 1 hour on a weekly basis for 15-20 sessions. (Extensive time and resources imply important difficulties for the application of this type of therapy.) For these reasons, and in an effort to reduce the high medical costs of depression treatment and overcome the difficulties of traditional treatments in primary care, brief psychotherapy for depression effective and cost-effective internet-based interventions have been extensively developed during the last decade [11,12]. Low-intensity, internet-based interventions could be a simple, cost-effective method for treating depression in primary care settings [13]. In fact, meta-analyses suggest that depression can be effectively treated with brief psychotherapy (6-8 sessions), specifically with cognitive behavioral therapy (CBT), problem-solving therapy [14], and counseling approaches [15].

Most of the internet interventions aiming at the treatment of depression are based on CBT. Previous findings for other forms of face-to face psychotherapy suggest that there is no *one-fits-all* solution [16], but few studies have analyzed web-based interventions based on other types of treatments. First results are promising, but more research is needed to determine the efficacy of alternative programs than internet-delivery CBT [17].

In a previous study, our group shows the efficacy of an internet intervention for depression in primary care (smiling is fun) [18]. The program follows a transdiagnostic perspective, and it is based on CBT techniques but also includes other psychological strategies to improve depressive symptoms such as promotion of healthy lifestyles, positive affect, and mindfulness. The treatment protocol is composed of 10 modules and lasts about 3 months. The program proved to be more effective than treatment as usual alone, but the attrition rate at follow-up was significant and the retention rate was not as good as we expected. These results led us to design a new protocol with the objective to identify which of the alternative therapeutic approaches was more effective and, also, to shorten the duration of the program to achieve better rates of attrition and retention.

Evidence of the benefits for treating depression of positive psychology, mindfulness, and lifestyle habits delivered using internet is growing as a result of increase in research studies over the last 10 years [19-22]. Meta-analyses of positive psychological interventions delivered to clinical and community samples have reported small but significant effects for reducing depressive symptoms [23] and comparable efficacy and lasting effects to traditional psychotherapy and pharmacotherapy [24,25]. Web-based positive emotion skills training for depression have shown preliminary good results [19,26] but warrant additional study. There is growing evidence for positive benefits of mindfulness interventions in clinically depressed individuals [27,28]. Recent reviews and meta-analysis of mindfulness-based CBT suggest that internet-based approaches have potential to contribute to improving depression [29,30], but there is a need for research into this therapeutic approach. Finally, lifestyle web-based interventions can be an effective and inexpensive alternative or supplement to depression therapy that is delivered using more traditional modes, overcoming barriers that make people from accessing treatment difficult. The results of a recent review [31] highlight the potential of web-based lifestyle interventions as adjunctive treatments for depression and the possibility of achieving significant improvements in depressive symptoms when targeting lifestyle behavior change. But the limited number of studies requires further clinical trials to achieve better understanding.

### Objectives

Considering the scarcity of these studies and the fact that low-intensity, internet-based psychological interventions could be an efficacious and cost-effective therapeutic option for the treatment of depression, the aim of this study was to assess the effectiveness of 3 low-intensity, internet-based psychological interventions (psychoeducational program for the promotion of a healthy lifestyle (HLP), psychological intervention for the promotion of positive affect (PAPP), and brief intervention based on mindfulness [MP]) compared with a control condition.

## Methods

### Study Design

This study was a multicenter, 4-arm, parallel randomized controlled trial. Adults with depressive symptoms in primary care were randomly assigned to one of the following groups: (1) HLP + improved treatment as usual (iTAU), (2) PAPP + iTAU, (3) MP + iTAU, or (4) iTAU.

Trial registration number of this study was ISRCTN82388279. Research protocol of the study has been described elsewhere [32].

### Recruitment of Participants and Baseline Assessment

We recruited patients with major depression or dysthymia, older than 18 years, able to understand and read Spanish, with mild or moderate depression according to the Patient Health Questionnaire-9 (PHQ-9; 5-9: mild depression; 10-14: moderate depression) [33], and with symptoms lasting longer than 2 weeks. Major depression and dysthymia were identified using the MINI International Neuropsychiatric Interview 5.0. We excluded patients with a diagnosis of any disease that may affect the central nervous system (brain pathology, traumatic brain injury, dementia, etc); with any psychiatric disorder other than major depression, dysthymia, anxiety disorders, or personality disorders; with any medical, infectious, or degenerative disease that may affect mood; with presence of delusional ideas or hallucinations consistent or not with mood; and with suicide risk.

Participants were recruited in primary care settings, between March 2015 and March 2016, in the Spanish regions of Aragon, Andalusia, and the Balearic Islands. When the general practitioner identifies a potential participant during a routine visit, he or she explained to the patient the characteristics of the study. When the patient was interested in participating, he or she signed an informed consent form and the general practitioner filled a referral form describing the sociodemographic characteristics of the patient and a checklist for inclusion and exclusion criteria and gave him or her the patient’s information sheet and a handout describing the study. The general practitioner sent these documents by fax to the local researcher. Participants were interviewed in the next 3 days by the researcher, which administered psychological assessment instruments related with inclusion and exclusion criteria by phone. Included participants were randomized to 1 of the 4 groups by an independent researcher. Patient safety was systematically monitored. The Ethical Review Board of the regional health authority approved the study (Ref: IB 2144/13PI).

### Randomization, Concealment, and Blinding

The sequence was concealed until interventions were assigned. Participants agreed to participate before the random allocation without knowing which treatment they were being allocated to. Study personnel conducting psychological assessment were masked to participants’ treatment conditions. The researcher that administered baseline assessments was unaware of the treatment group to which the participant belonged. This researcher was different from the one that administered the questionnaires over the study. General practitioners were also unaware, as far as possible, of the arm to which each patient had been randomized, as their treatment needed to be exclusively based on the recommendations of the treatment of depression guidelines.

### Follow-Up

Follow-up data collection took place between April 2015 and June 2017. Participants were assessed on web at pretreatment (time 1), posttreatment (time 2), and 6- (time 3) and 12- (time 4) month posttreatment assessments. The web-based platform hosted the questionnaires. Participants were sent an email with a link to that platform. No other protocols were used to increase compliance with the research data collection, but a phone call was made before each wave assessment to increase response rates.

### Improved Treatment as Usual

All the patients included in the study (irrespective of the treatment group randomly assigned) received iTAU. This treatment was provided by their general practitioners, who had previously received a training program to update their knowledge on how to diagnose and treat depression in primary care and optimized by the recommendations based on the Spanish Guide for the Treatment of Depression in Primary Care [34,35]. In case of suicide risk or severe social dysfunction or worsening of symptoms being detected, patients were referred to mental health facilities.

### Intervention Groups

All interventions (except iTAU) were composed of one face-to-face group session and 4 web-based, individual, and interactive therapeutic modules.

The face-to-face session, which took place in primary care centers, involved up to 5 patients and was 90 min long. The aim of this session was to explain the program structure and main components of treatment and to motivate participants for change.

The web-based therapeutic modules are oriented to work on different psychological techniques, and the duration of each module is approximately between 40 and 60 min. All modules include an explanation of the module contents, check questions to test if they understand the contents, and exercises to practice the techniques. These modules are sequential, to move step by step, throughout the program. However, users can review the module contents once they are finished. Although the duration of the program can vary among users, it is estimated that for most people, it lasted between 4 and 8 weeks. Regarding the therapeutic content, all intervention groups are composed of 4 intervention modules based on different psychological techniques, as shown in Table 1. A more specific and detailed description of the module contents can be found elsewhere [32].

To maximize adherence, participants received 2 weekly automated mobile phone messages, encouraging them to proceed with the program and reminding them of the importance of doing the tasks in each module. If participants did not access the program for a week, they received an automated email encouraging them to continue with the modules. Furthermore, the program also offers continued feedback to users through the assessment tools showing them their progress throughout the entire treatment process. All groups of patients received a participant manual with information about the technical aspects of the web-based program.

**Table 1:** Intervention modules and main objectives.

Intervention and modules		Main objective
**Psychoeducational program for the promotion of a healthy lifestyle**		
	Beginning of a lifestyle change	To teach the importance of healthy lifestyle to improve emotional health and general well-being and to give structured hygienic-dietary recommendations.
	Physical activity. Learning to move on	To give information about the most recommended exercises to improve mood, and to train the patient in learning procedures to increase motivation, to start being more active, and to maintain this physical activity regularly.
	Diet. Learning to eat	To teach the importance of diet to achieve a good physical and mental health, and the role of the Mediterranean diet in the prevention and treatment of depression.
	Sleep. The importance of good sleep	To understand the relationship between sleep and general health.
**Psychological intervention for the promotion of positive affect**		
	Learning to live	To teach the importance of establishing and maintaining an adequate activity level and the relevance of choosing activities that are significant, with a personal meaning for the individual.
	Learning to enjoy	To give education about the effect of positive emotions and to train the patient in learning procedures to increase the likelihood of experiencing positive emotions, promoting the occurrence of pleasant activities to learn to enjoy the present moment.
	Accepting to life	To train the patient in focusing on positive emotions related with the past (such as gratitude) or the future (such as optimism).
	Living and learning	To train the patient in understanding life as a continuous process of learning and personal growth, emphasizing the training in strategies to promote psychological strengths, resilience, and meaningful goals linked to important values.
**Brief intervention based on mindfulness**		
	Getting to know mindfulness	To show what mindfulness is, prejudices about it, the inattention problem, and some of its main benefits and recommendations to practice it.
	Establishing formal and informal practices	To teach the importance of the establishment not only of formal but also of informal practice.
	Through management, body scan practice and values	To help people to see the importance of values to keep a regular mindfulness practice.
	Self-compassion. Integrating mindfulness in everyday life	To establish a regular practice of mindfulness to be indefinitely kept.

### Instruments

#### Demographic Variables

We gathered sociodemographic data such as gender, age, place of residence, family status, living with family or alone, level of studies, work status, and income level according to national minimum wage (NMW) as well as clinical variables such as taking psychopharmacological medication (yes vs no) and the number of general practitioners visits in the previous 12 months.

#### Outcomes

The Spanish version of PHQ-9 [36], as a continuous variable, was used as the primary outcome measure in all 4 assessments, from pretreatment (time 1) to 12-month follow-up (time 4). PHQ-9 is one of the most widely used instruments to evaluate the presence and severity of depressive symptoms. Participants describe their mood according to the last 2 weeks before evaluation. Items range from 0 to 3 denoting *not at all*, *several days*, *more than half the days*, and *nearly every day*, respectively. Total scores range from 0 to 27. The Spanish version has been shown to have good psychometric properties (for the diagnosis of any disorder, k=0.74; overall accuracy, 88%; sensitivity, 87%; and specificity, 88%).

Secondary outcomes included the visual analog scale (VAS) of the EuroQol (*EuroQol—a new facility for the measurement of health-related quality of life*, 1990), in its Spanish version [37]; the Short-Form Health Survey (SF-12) [38], in its Spanish version [39], as a measure of health-related quality of life and functioning; the Positive and Negative Affect Schedule (PANAS) [40], in its Spanish version [41], as a measure of positive and negative affect; and the Pemberton Happiness Index (PHI) [42] as a measure of general well-being.

The VAS is a vertical line on which the best and worst possible health states are scored, 100 or 0, respectively. The SF-12 scoring algorithm yields a physical and mental component scale, and both were used as continuous variables applying Spanish norms. The PANAS evaluates 2 independent dimensions: positive affect and negative affect and were used as continuous variables. *Trait* version was used in this study. The overall PHI index was used as a continuous measure of general well-being, and the scores ranged from 0 to 10.

#### Sample Size

Required sample size was 240 participants, 60 participants in each condition [32]. This estimation was calculated according to the literature, with a SD of 9.2 and a mean of 16.2 in the iTAU group [43], 14.59 in PAPP group [44], 16.12 in HLP group [45], and 10.3 in the MP group [43], accepting an α of .05 and a β risk<0.2 in a bilateral contrast and assuming a 25.0% (60/240) patient loss to follow-up. This sample size also allows for the calculation of the clinically significant difference in the main outcome variable, PHQ-9 [36], and this difference has been placed at 5 points.

#### Data Analysis

First, demographic and outcome variables were characterized through descriptive exploratory analysis. Database scrutiny revealed increasing percentages of whole wave missingness in primary and secondary outcome variables along the follow-up. Missingness effects were, thereafter, assessed through sensitivity analyses for demographic variables, intervention groups, and baseline outcomes, considering dropout as study abandonment, with or without subject return, at any assessment period. Although an association between collected variables and study attrition had been detected, no association was reported between outcome values and follow-up missingness; hence, missing at random was assumed for primary and secondary outcome variables. Finally, we implemented Multiple Imputation with Chained Equations (MICE) to replace the outcome missing values, performing 100 imputation models with 100 iterations per model.

We conducted paired *t*-tests and Wilcoxon Signed Rank tests to estimate PHQ-9 primary outcome differences between study stages. Analysis of variance and Tukey’s range test were also displayed to examine outcome differences between intervention groups at each time point. In addition, unadjusted and adjusted to sex and age linear regression models were performed. Consecutively, Hedge’s (g) effect size index was calculated for each unadjusted regression model. The same approach was used compare secondary results from SF-12 Mental and Physical subscales scores, EuroQol (VAS) scores, and PHI global scores. We used a Complier Average Causal Effect (CACE) analysis to determine the number of completed modules effect on PHQ-9 posttreatment scores, defining compliance as 4 completed modules (100%) and analyzing these same effects per module. In all the analyses, we used a 2-sided test at 5% significance level. Data analyses were implemented with R (3.15.) and Stata 15 (StataCorp).

## Results

### Baseline Characteristics and Attrition Rates

**Figure 1:**
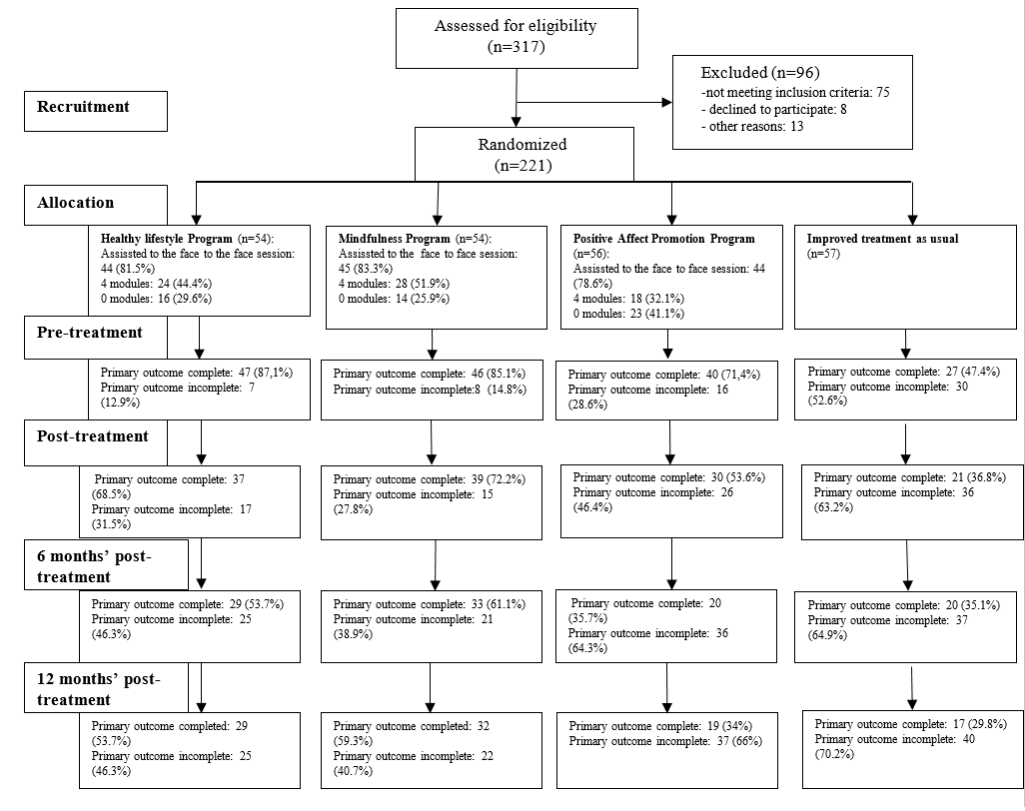
Flow diagram.

A total of 221 recruited participants met inclusion criteria and agreed to participate after baseline assessments (Figure 1). The number of recruited participants varied across regions: 75 from Zaragoza, 76 from Málaga, and 70 from Mallorca. A total of 57 participants were designated to iTAU, 54 to HLP, 54 to MP, and the remaining 56 to PAPP. Although all participants did not provide their complete baseline sociodemographic data, no statistically significant differences were found between intervention groups after randomization (Table 2).

Attrition rates increased significantly as study went forward: primary outcome PHQ-9 data were collected for the 72.4% (160/221) of participants at time 1, 57.5% (127/221) at time 2, 46.2% (102/221) at time 3, and 43.9% (97/221) at time 4. Missingness analysis does not report outcome baseline significant differences between dropout and nondropout groups. Conversely, differences in missingness were found between intervention groups, with 28% (15/54) in HLP and 24% (13/54) in MP presenting significative less dropout subjects (*P*<.01) than 45% (25/56) in PAPP and 63% (36/57) in iTAU groups. Baseline age differences were also detected between dropout and nondropout groups at time 2 (*P*=.04) and time 3 (*P*=.03). After MICE, mean depression severity assessed by PHQ-9 score at pretreatment stage (time 1) was 15.33 (SD 5.76) with a median of 15.5, which agrees for a moderate depression level.

**Table 2:** Baseline characteristics of participants between intervention groups.

Intervention characteristics and measures			Intervention groups			
			iTAU^a^	HLP^b^	MP^c^	PAPP^d^
**Sociodemographic characteristics**						
	Age, mean (SD)		44.54 (16.10)	44.67 (9.98)	47.50 (13.09)	44.53 (10.23)
	Sex (female), n (%)		41 (72)	40 (74)	47 (87)	44 (79)
	Married, n (%)		24 (52)	23 (47)	27 (59)	31 (62)
	Living with family or couple, n (%)		38 (83)	36 (75)	34 (76)	44 (88)
	High education, n (%)		14 (33)	18 (45)	17 (43)	19 (40)
	**Employed, income level, n (%)**		20 (44)	22 (47)	17 (40)	25 (50)
		<1 NMW^e^	6 (19)	10 (30)	7 (23)	13 (33)
		1-2 NMW	16 (52)	16 (48)	7 (23)	14 (36)
		2-4 NMW	8 (26)	7 (21)	12 (40)	12 (31)
		>4 NMW	1 (3)	0 (0)	4 (13)	0 (0)
**Clinical measures**						
	**Depression severity**					
		PHQ-9^f^, mean (SD); median (IQR)	12.46 (2.10); 13.0 (11-14)	12.57 (2.46); 13.5 (11-14)	12.67 (2.56); 13.0 (11-14)	12.63 (2.03); 13.0 (11.5-14)
	**Perceived health**					
		Physical SF-12^g^, mean (SD); median (IQR)	43.06 (11.03); 41.38 (35.49-51.89)	42.16 (10.66); 39.84 (35.07-52.02)	42.52 (9.75); 42.11 (35.58-48.27)	45.17 (13.54); 45.74 (35.04-55.65)
		Mental SF-12, mean (SD); median (IQR)	26.75 (9.62); 24.54 (20.97-30.58)	27.59 (9.61); 25.98 (21.47-32.75)	26.96 (10.86); 24.51 (20.93-28.66)	26.22 (9.97); 23.79 (19.77-29.85)
		VAS EuroQol^h^, mean (SD); median (IQR)	51.60 (17.95); 50 (40-60)	52.22 (21.63); 50 (30-70)	48.91 (26.01); 50 (30-70)	49.00 (19.05); 50 (40-60)
		Overall PHI^i^ index, mean (SD); median (IQR)	4.3 (1.86); 4.33 (2.92-5.42)	4.4 (1.95); 4.42 (3-5.42)	4.32 (1.83); 4.42 (2.96-5.17)	4.25 (1.98); 4.58 (2.83-5.42)
		Positive affect PANAS^j^, mean (SD); median (IQR)	18.56 (6.43); 17 (14-23)	19.04 (6.94); 18 (13-25)	19.48 (6.60); 18 (15-22)	18.09 (6.00); 17 (13-22)
		Negative affect PANAS, mean (SD); median (IQR)	28.27 (8.45); 27 (22-34)	28.91 (8.34); 29 (23-35)	27.89 (8.07); 27 (22-32.75)	29.46 (8.79); 27 (23-35.5)

^a^iTAU: improved treatment as usual.

^b^HLP: healthy lifestyle program.

^c^MP: mindfulness program.

^d^PAPP: positive affect promotion program.

^e^NMW: national minimum wage.

^f^PHQ-9: Patient Health Questionnaire-9 items.

^g^SF-12: 12-item Short-Form Health Survey.

^h^VAS EuroQol: visual analog scale of the EuroQol.

^i^PHI: Pemberton Happiness Index.

^j^PANAS: Positive and Negative Affect Schedule.

### Primary Analysis

All our primary and secondary results were extracted from databases with high rates of attrition, 44.7% (395/884), which were thereafter imputed. Thus, the following results should be considered more as suggestive hypothesis rather than empirical statements. To this extent, we found significant decreases of PHQ-9 scores (*P*<.001) in all interventions (iTAU included) from pretreatment (mean 15.33, SD 5.76) to posttreatment (mean 10.19, SD 6.42) and up to 6 and 12 months (time 3: mean 9.39, SD 6.59 and time 4: mean 9.68, SD 6.14). After treatment, a moderate decrease was detected in PHQ scores from HLP and MP relative to iTAU: iTAU versus HLP (β=–3.05; *P*=.01) and iTAU versus MP (β=–3.00; *P*=.01; Table 3). In contrast, we observed no significant PHQ-9 differences between PAPP and iTAU, nor between any psychotherapeutic strategies, throughout the study (Table 3). Adjusted regression models replicated these same findings with poor variation (Multimedia Appendix 1). CACE analysis reported posttreatment dose-response significant decrease in PHQ-9 scores in HLP and PAPP in both imputed and adjusted models (Table 4, Multimedia Appendix 2). Despite the fact that the compliance effects (4 modules vs >4 modules) tended to disappear in the long term, the effects per module remained conserved (Table 4).

**Table 3:** Primary outcome analysis with imputed data (N=221): intervention comparisons along the follow-up.

Primary outcome			Time 1 (pretreatment)		Time 2 (posttreatment)		Time 3 (6 months)		Time 4 (12 months)	
**PHQ-9^a^**										
	**iTAU^b^** **vs HLP^c^**									
		g^d^		0.25		–0.50		–0.22		–0.06
		*P* value		.20		*.01* ^e^		.23		.73
		β^f^ (95% CI)		1.41 (–0.75 to 3.57)		–3.05 (–5.43 to –0.68)		–1.52 (–3.98 to 0.95)		–0.40 (–2.71 to 1.91)
	**iTAU vs MP^g^**									
		g		–0.17		0.47		0.24		–0.01
		*P* value		.36		.*01*		.18		.96
		β (95% CI)		1.00 (–1.16 to 3.16)		–3.00 (–5.37 to –0.63)		–1.68 (–4.15 to 0.78)		0.06 (–2.25 to 2.37)
	**iTAU vs PAPP^h^**									
		g		–0.23		0.23		0.31		0.01
		*P* value		.20		.22		.10		.99
		β (95% CI)		1.40 (–0.74 to 3.54)		–1.46 (–3.81 to 0.89)		–2.08 (–4.52 to 0.37)		–0.02 (–2.31 to 2.27)
	**HLP vs MP**									
		g		0.07		-0.01		0.03		–0.07
		*P* value		.71		.96		.90		.70
		β (95% CI)		0.41 (–1.78 to 2.57)		–0.06 (–2.46 to 2.35)		0.17 (–2.33 to 2.67)		–0.46 (–2.81 to 1.88)
	**HLP vs PAPP**									
		g		0.00		–0.25		0.09		–0.06
		*P* value		.99		.19		.66		.75
		β (95% CI)		0.01 (–2.16 to 2.18)		–1.59 (–3.97 to 0.79)		0.56 (–1.92 to 3.04)		–0.38 (–2.70 to 1.94)
	**MP vs PAPP**									
		g		–0.07		–0.23		0.06		0.01
		*P* value		.72		.21		.76		.94
		β (95% CI)		0.40 (–1.77 to 2.57)		1.53 (–0.85 to 3.92)		–0.39 (–2.87 to 2.08)		–0.08 (–2.41 to 2.24)

^a^PHQ-9: Patient Health Questionnaire-9 items.

^b^iTAU: improved treatment as usual.

^c^HLP: healthy lifestyle program.

^d^g: Hedge’s effect size measure*.*

^e^Statistically significant values (*P*<.05) are shown in italics.

^f^β: regression coefficient.

^g^MP: mindfulness program.

^h^PAPP: positive affect promotion program.

**Table 4:** Dose-response in imputed primary outcome at posttreatment and along the follow-up.

Interventions			Pretreatment to posttreatment				Pretreatment to posttreatment				Pretreatment to posttreatment		
			β^a^ (95% CI)		*P* value		β (95% CI)		*P* value		β (95% CI)		*P* value
**HLP^b^**													
	CACE^c^ analysis^d^	–3.32 (4.43 to –2.21)		*.004* ^e^		–0.51 (2.03 to 1.02)		.74		–1.03 (2.61 to 0.56)		.52	
	Effect per session	–0.28 (0.34 to –0.21)		*.001*		–0.17 (0.25 to –0.09)		*.03*		–0.06 (0.13 to 0.02)		.45	
**MP^f^**													
	CACE analysis	0.93 (0.71 to 2.57)		.57		1.01 (0.72 to 2.74)		.56		1.13 (0.5 to 2.76)		.49	
	Effect per session	0.26 (0.22 to 0.74)		.59		–0.17 (0.25 to –0.09)		*.03*		–0.06 (0.13 to 0.02)		.45	
**PAPP^g^**													
	CACE analysis	0.24 (1.30 to 1.78)		.88		0.96 (0.66 to 2.57)		.56		0.30 (1.24 to 1.84)		.85	
	Effect per session	–0.28 (0.34 to –0.21)		.*001*		–0.17 (0.25 to –0.09)		*.03*		–0.06 (0.13 to 0.02)		.45	
**All**													
	CACE analysis	–2.11 (2.86 to –1.36)		*.006*		–0.49 (1.37 to 0.4)		.58		–0.22 (1.04 to 0.6)		.79	
	Effect per session	–0.28 (0.34 to –0.21)		*.001*		–0.17 (0.25 to –0.09)		*.03*		–0.06 (0.13 to 0.02)		.45	

^a^β: regression coefficients.

^b^HLP: healthy lifestyle program.

^c^CACE: Complier Average Causal Effect.

^d^Compliance as attendance >4 modules.

^e^Statistically significant values (*P*<.05) are shown in italics.

^f^MP: mindfulness program.

^g^PAPP: positive affect promotion program..

### Secondary Analysis

Imputed Mental and Physical SF-12 scores significantly increased in all intervention groups (iTAU included) from pretreatment to posttreatment (Mental SF-12: *P*<.001; Physical SF-12: *P*<.001) and from posttreatment to 6 months after treatment (Mental SF-12: *P*<.001; Physical SF-12: *P*=.02). Although differences between these intervention groups disappeared in the long term (Table 5), iTAU Mental SF-12 scores were higher than HLP scores (β=–5.32; *P=*.02) and PAPP scores (β=–7.72; *P=*.001) at posttreatment. Conversely, we determined posttreatment increases in HLP Physical SF-12 scores relative to iTAU (β=4.58; *P=*.047) and from MP group compared with iTAU (β=5.32; *P=*.02). Physical SF-12 HLP, MP, and PAPP group differences relative to iTAU were observed up to 6 months after treatment (Table 5). These results were replicated similarly by adjusted regression coefficients (Multimedia Appendix 3).

Although EuroQol (VAS) significant differences were detected from pretreatment to 6 months (*P*<.001) and up to 12 months after treatment (*P*<.001) in all intervention groups, no meaningful differences among the groups were observed at any time (Table 6). Otherwise, PHI scores rose significantly at 6 and 12-months after treatment relative to pretreatment (*P*<.001), and all psychotherapy interventions, except HLP, reported better PHI results than iTAU treatment at time 3 (Table 6). These results were replicated similarly by adjusted regression coefficients (Multimedia Appendix 4). PANAS negative affect scale decreased in all intervention groups at posttreatment (*P*<.001), at 6 months (*P*<.001), and at 12 months (*P*<.001) compared with pretreatment. In contrast, all PANAS positive affect scores increased significantly throughout study time relative to pretreatment (*P*<.001). However, we found no significant PANAS positive affect differences between intervention groups throughout the study (Table 7). Regarding PANAS negative scale, only PAPP intervention was significantly lower than iTAU when were compared 12 months after treatment (Table 7). These results were replicated similarly by adjusted regression coefficients (Multimedia Appendix 5).

**Table 5:** Short-Form Health Survey-12 (Mental and Physical) outcome analysis with imputed data (N=221): intervention comparisons along the follow-up.

Secondary outcomes			Time 1 (pretreatment)		Time 2 (posttreatment)		Time 3 (6 months)		Time 4 (12 months)
**Mental Scale SF-12^a^**									
	**iTAU^b^** **vs HLP^c^**								
		g^d^	–0.06		–0.42		–0.11		0.01
		*P* value	.73		.*02*^e^		.53		.98
		β^f^ (95% CI)	–0.63 (–4.25 to 2.99)		–5.32 (–9.91 to –0.72)		–1.53 (–6.30 to 3.23)		–0.05 (–5.04 to 4.93)
	**iTAU vs MP^g^**								
		g	0.14		0.16		0.01		–0.05
		*P* value	.46		.38		.94		.81
		β (95% CI)	–1.37 (–4.99 to 2.24)		–2.06 (–6.66 to 2.53)		–0.18 (–4.95 to 4.58)		0.62 (–4.36 to 5.61)
	**iTAU vs PAPP^h^**								
		g	0.15		0.67		0.22		0.24
		*P* value	.45		*.001*		.28		.20
		β (95% CI)	–1.37 (–4.96 to 2.21)		–7.72 (–12.27 to –3.16)		–2.61 (–7.33 to 2.11)		–3.2 (–8.14 to 1.74)
	**HLP vs MP**								
		g	0.07		–0.25		–0.1		–0.05
		*P* value	.69		.17		.58		.79
		β (95% CI)	–0.74 (–4.41 to 2.93)		3.25 (–1.41 to 7.91)		1.35 (–3.48 to 6.18)		0.68 (–4.37 to 5.73)
	**HLP vs PAPP**								
		g	0.01		0.17		–0.07		–0.12
		*P* value	.97		.38		.72		.49
		β (95% CI)	–0.07 (–4.22 to 4.08)		–2.04 (–6.59 to 2.51)		0.76 (–3.41 to 4.92)		1.36 (–2.51 to 5.24)
	**MP vs PAPP**								
		g	0.23		0.23		0.25		0.09
		*P* value	.20		.23		.23		.62
		β (95% CI)	–2.72 (–6.87 to 1.43)		–2.77 (–7.32 to 1.78)		–2.53 (–6.69 to 1.64)		–0.97 (–4.85 to 2.9)
**Physical Scale SF-12**									
	**iTAU vs HLP**								
		g	0.04	0.37		0.39		–0.19	
		*P* value	.86	.*047*		*.03*		.35	
		β (95% CI)	0.36 (–3.77 to 4.5)	4.58 (0.05 to 9.11)		4.73 (0.58 to 8.87)		–1.85 (–5.7 to 2.01)	
	**iTAU vs MP**								
		g	–0.25	–0.42		–0.73		–0.05	
		*P* value	.15	*.02*		.*001*		.80	
		β (95% CI)	3.01 (–1.12 to 7.14)	5.32 (0.79 to 9.85)		8.01 (3.87 to 12.16)		0.49 (–3.37 to 4.35)	
	**iTAU vs PAPP**								
		g	–0.03	–0.21		–0.50		0.05	
		*P* value	.89	.27		*.009*		.80	
		β (95% CI)	0.29 (–3.8 to 4.39)	2.54 (–1.94 to 7.03)		5.49 (1.38 to 9.6)		–0.48 (–4.3 to 3.34)	
	**HLP vs MP**								
		g	–0.22	–0.06		–0.29		–0.21	
		*P* value	.21	.75		.13		.24	
		β (95% CI)	2.65 (–1.54 to 6.83)	0.73 (–3.86 to 5.32)		3.28 (–0.92 to 7.49)		2.34 (–1.57 to 6.25)	
	**HLP vs PAPP**								
		g	0.08	0.20		0.09		0.23	
		*P* value	.69	.31		.66		.22	
		β (95% CI)	–0.74 (–4.37 to 2.9)	–2.4 (–7.02 to 2.22)		–1.08 (–5.86 to 3.71)		–3.15 (–8.15 to 1.86)	
	**MP vs PAPP**								
		g	0.01	0.47		0.20		0.28	
		*P* value	>.99	.*02*		.32		.13	
		β (95% CI)	0.01 (–3.63 to 3.64)	–5.65 (–10.27 to –1.03)		–2.43 (–7.21 to 2.36)		–3.82 (–8.83 to 1.18)	

^a^SF-12: 12-item Short-Form Health Survey.

^b^iTAU: improved treatment as usual.

^c^HLP: healthy lifestyle program.

^d^g: Hedge’s effect size measure*.*

^e^Statistically significant values (*P*<.05) are shown in italics.

^f^β: regression coefficient.

^g^MP: mindfulness program.

^h^PAPP: positive affect promotion program.

**Table 6:** Visual analog scale of the EuroQol and Pemberton Happiness Index outcome analysis with imputed data (N=221): intervention comparisons along the follow-up.

Secondary outcomes			Time 1 (pretreatment)	Time 3 (6 months)	Time 4 (12 months)
**VAS EuroQol^a^**					
	**iTAU^b^** **vs HLP^c^**				
		g^d^	0.02	–0.20	–0.33
		*P* value	.91	.31	.08
		β^e^ (95% CI)	0.47 (–7.93 to 8.87)	–3.82 (–11.17 to 3.53)	–6.56 (–13.86 to 0.75)
	**iTAU vs MP^f^**				
		g	0.17	0.18	0.16
		*P* value	.33	.33	.39
		β (95% CI)	–4.16 (–12.56 to 4.24)	–3.63 (–10.98 to 3.72)	–3.17 (–10.47 to 4.14)
	**iTAU vs PAPP^g^**				
		g	0.18	0.10	0.09
		*P* value	.36	.60	.63
		β (95% CI)	–3.9 (–12.22 to 4.43)	–1.95 (–9.23 to 5.33)	–1.76 (–9.00 to 5.48)
	**HLP vs MP**				
		g	0.19	–0.01	–0.17
		*P* value	.29	.96	.58
		β (95% CI)	–4.63 (–13.14 to 3.88)	0.19 (–7.26 to 7.63)	–2.04 (–9.21 to 5.2)
	**HLP vs PAPP**				
		g	0.21	–0.10	–0.24
		*P* value	.31	.62	.20
		β (95% CI)	–4.37 (–12.8 to 4.07)	1.86 (–5.52 to 9.24)	4.79 (–2.55 to 12.13)
	**MP vs PAPP**				
		g	–0.01	–0.08	–0.07
		*P* value	.95	.65	.71
		β (95% CI)	0.26 (–8.17 to 8.7)	1.68 (–5.7 to 9.06)	1.4 (–5.93 to 8.74)
**PHI^h^**					
	**iTAU vs HLP**				
		g	–0.05	0.19	–0.18
		*P* value	.81	.30	.33
		β (95% CI)	–0.09 (–0.79 to 0.61)	0.40 (–0.35 to 1.16)	–0.32 (–0.98 to 0.33)
	**iTAU vs MP**				
		g	0.08	–0.49	–0.06
		*P* value	.65	.*01*^i^	.75
		β (95% CI)	–0.16 (–0.86 to 0.54)	0.98 (0.22 to 1.73)	0.11 (–0.55 to 0.77)
	**iTAU vs PAPP**				
		g	0.10	–0.42	0.09
		*P* value	.59	.*03*	.61
		β (95% CI)	–0.19 (–0.89 to 0.5)	0.82 (0.07 to 1.57)	–0.17 (–0.82 to 0.48)
	**HLP vs MP**				
		g	0.04	–0.27	–0.25
		*P* value	.84	.14	.20
		β (95% CI)	–0.07 (–0.78 to 0.64)	0.57 (–0.19 to 1.34)	0.43 (–0.23 to 1.1)
	**HLP vs PAPP**				
		g	0.06	–0.2	–0.08
		*P* value	.77	.28	.65
		β (95% CI)	–0.11 (–0.81 to 0.6)	0.42 (–0.34 to 1.17)	0.15 (–0.51 to 0.81)
	**MP vs PAPP**				
		g	0.02	0.08	0.16
		*P* value	.93	.68	.41
		β (95% CI)	–0.03 (–0.74 to 0.67)	–0.16 (–0.92 to 0.6)	–0.28 (–0.94 to 0.38)

^a^VAS EuroQol: visual analog scale of the EuroQol.

^b^iTAU: improved treatment as usual.

^c^HLP: healthy lifestyle program.

^d^g: Hedge’s effect size measure*.*

^e^β: regression coefficient.

^f^MP: mindfulness program.

^g^PAPP: positive affect promotion program.

^h^PHI: Pemberton Happiness Index.

^i^Statistically significant values (*P*<.05) are shown in italics.

**Table 7:** Positive and Negative Affect Scales outcome analysis with imputed data (N=221): intervention comparisons along the follow-up.

Secondary outcomes				Time 1 (pretreatment)		Time 2 (posttreatment)		Time 3 (6 months)		Time 4 (12 months)
**Positive Scale PANAS^a^**										
	**iTAU^b^** **vs HLP^c^**									
		g^d^	0.07		–0.01		0.25		0.34	
		*P* value	.72		.94		.17		.07	
		β^e^ (95% CI)	0.45 (–1.96 to 2.85)		–0.12 (–3.47 to 3.23)		2.24 (–1.00 to 5.48)		3.36 (–0.30 to 7.01)	
	**iTAU vs MP^f^**									
		g	0.14		0.00		–0.16		–0.33	
		*P* value	.44		>.99		.41		.09	
		β (95% CI)	–0.94 (–3.35 to 1.46)		–0.01 (–3.36 to 3.34)		1.35 (–1.88 to 4.59)		3.19 (–0.46 to 6.85)	
	**iTAU vs PAPP^g^**									
		g	0.21		0.31		0.05		–0.15	
		*P* value	.25		.10		.81		.41	
		β (95% CI)	–1.41 (–3.79 to 0.97)		–2.78 (–6.09 to 0.54)		–0.40 (–3.61 to 2.81)		1.51 (–2.11 to 5.13)	
	**HLP vs MP**									
		g	0.22		–0.01		0.10		0.02	
		*P* value	.26		.95		.59		.93	
		β (95% CI)	–1.39 (–3.83 to 1.05)		0.11 (–3.28 to 3.5)		–0.89 (–4.17 to 2.39)		–0.17 (–3.87 to 3.54)	
	**HLP vs PAPP**									
		g	0.29		0.31		0.3		0.19	
		*P* value	.13		.12		.11		.32	
		β (95% CI)	–1.86 (–4.27 to 0.56)		–2.66 (–6.02 to 0.71)		–2.64 (–5.89 to 0.61)		–1.85 (–5.52 to 1.82)	
	**MP vs PAPP**									
		g	0.08		0.33		0.21		0.17	
		*P* value	.70		.11		.29		.37	
		β (95% CI)	–0.47 (–2.88 to 1.95)		–2.77 (–6.13 to 0.59)		–1.75 (–5.00 to 1.5)		–1.68 (–5.35 to 1.99)	
**Negative Scale PANAS**										
	**iTAU vs HLP**									
		g	–0.12		–0.32		–0.16		–0.36	
		*P* value	.52		.09		.39		.07	
		β (95% CI)	–1.02 (–4.18 to 2.14)		–2.81 (–6.04 to 0.42)		–1.37 (–4.51 to 1.78)		–3.15 (–6.51 to 0.21)	
	**iTAU vs MP**									
		g	–0.06		0.31		0.16		0.24	
		*P* value	.73		.10		.40		.18	
		β (95% CI)	0.55 (–2.61 to 3.71)		–2.73 (–5.96 to 0.5)		–1.35 (–4.49 to 1.80)		–2.32 (–5.67 to 1.04)	
	**iTAU vs PAPP**									
		g	0.25		0.31		0.33		0.41	
		*P* value	.19		.11		.11		*.03* ^h^	
		β (95% CI)	–2.09 (–5.22 to 1.04)		–2.59 (–5.79 to 0.61)		–2.54 (–5.65 to 0.57)		–3.63 (–6.96 to –0.31)	
	**HLP vs MP**									
		g	–0.19		–0.01		0.00		–0.09	
		*P* value	.33		.96		.99		.63	
		β (95% CI)	1.57 (–1.63 to 4.78)		0.07 (–3.20 to 3.35)		0.02 (–3.17 to 3.20)		0.83 (–2.57 to 4.23)	
	**HLP vs PAPP**									
		g	0.13		–0.03		0.14		0.06	
		*P* value	.51		.90		.46		.78	
		β (95% CI)	–1.07 (–4.24 to 2.11)		0.21 (–3.03 to 3.46)		–1.17 (–4.33 to 1.98)		–0.48 (–3.85 to 2.89)	
	**MP vs PAPP**									
		g	0.3		–0.02		0.15		0.14	
		*P* value	.10		.93		.46		.44	
		β (95% CI)	–2.64 (–5.82 to 0.53)		0.14 (–3.10 to 3.38)		–1.19 (–4.35 to 1.96)		–1.32 (–4.69 to 2.06)	

^a^PANAS: Positive and Negative Affect Schedule.

^b^iTAU: improved treatment as usual.

^c^HLP: healthy lifestyle program.

^d^g: Hedge’s effect size measure*.*

^e^β: regression coefficient.

^f^MP: mindfulness program.

^g^PAPP: positive affect promotion program.

^h^Statistically significant values (*P*<.05) are shown in italics.

### Internet-Based Program Usage

A total of 81.1% (133/164) participants attended the initial face-to-face session. In HLP, 44% (24/54) of participants completed all web-based modules, in MP, 52% (28/54), and in PAPP, 32% (18/56; χ^2^_2_=4.4; *P*=.11). In HLP, the median number of sessions completed was 2 (range: 0-4). In MP, the median number of sessions completed was 4 (range: 0-4), and in PAPP, 2 (range: 0-4). There were no significant differences between intervention groups in terms of sessions completed (*F*_2_=1.775; *P*=.17).

## Discussion

### Principal Findings

The main objective of our study was to examine the efficacy of 3 low-intensity, internet-based psychological interventions when compared with that of the control condition (iTAU) in primary care in Spain.

Our main finding was that there were differences in the short term in favor of internet-based psychological interventions, specifically HLP and MP. This finding is consistent with the literature that has shown that brief psychotherapy is efficacious for the treatment of depression in primary care [12] and is also in line with the previous literature that has demonstrated that internet-based intervention programs are effective for the treatment of depression [18,46-48]. However, no differences were found in depression severity between PAPP and iTAU. This finding is not in line with the previous literature that has shown that this psychotherapy is effective in reducing depression [49]. Moreover, there were no differences found in the medium and long term between intervention and control groups. This result differs from findings of previous systematic reviews and meta-analysis that have evidenced that electronic health interventions effectively reduce depressive symptoms [11,50,51]. However, differences between populations may affect the generalizability of these studies [52]. It has been argued that psychotherapy may be less effective in primary care than in other settings, mainly due to the considerable heterogeneity in the severity of symptoms and the lower motivation among patients in primary care settings [53]. Our findings also show the faster effect of our interventions, with moderate effect size at posttreatment. The lack of mid- and long-term efficacy could be explained by a floor effect. Care as usual is not a clearly defined treatment, entailing different interventions [52]. Given that the usual care was improved before the interventions and that pharmacological treatment were offered to all groups, no major effects can be expected when web-based interventions were given. Our results also show that there were no differences between interventions throughout the study regarding depressive symptomatology. This finding is in line with that of the study by Cuijpers et al [54] who found that all therapies are equally effective in the treatment of depression.

Furthermore, we found differences between the 3 interventions groups and control group (iTAU) regarding health-related quality of life. In particular, we observed short- and medium-term differences in favor of HLP and MP in physical health status, and in medium term for PAPP. These results demonstrated that the 3 low-intensity, internet-based psychological interventions in primary care are also effective in improving medium- and long-term quality of life. This finding is highly important, for, as is well known, depression is associated with serious disability and loss in quality of life [55,56], suggesting that it could be a useful tool to improve quality of life of patients with depression.

Differences were also found in well-being and affect between intervention and control groups. In particular, we observed differences in medium term regarding well-being in MP compared with that in iTAU. In PAPP, differences were found in medium and long term with regard to well-being and, in long term, with regard to negative affect in favor of psychotherapy. This finding shows us that although PAPP seems to be ineffective in reducing depressive symptomatology, it could be effective in improving health-related quality of life and well-being in patients with depression.

Regarding treatment adherence, our completion treatment rates were relatively low in each intervention group. Dropout treatment is common in internet intervention programs, although rates vary depending on the support provided along the intervention or the context [47]. Regarding primary care, our adherence rates are higher than those reported in a previous study, in which participants completing all treatment sessions did not exceed 20% [57].

A possible explanation could be that in our study, there was an initial face-to-face group session, in which the final goal was to reinforce commitment and adherence to treatment, as well as, to explain the program structure and main components of treatment, clarify the instructions for the use of the web-based platform, and motivate participants to change. Thus, perhaps these measures could have increased our completion rates.

### Limitations and Strengths

This trial presents several limitations, which should be mentioned. First, not all participants completed posttest measurements, and a high attrition rate at follow-up was found. Although missing values were corrected by using multiple imputations, the results should be interpreted with caution. Second, just as difficulties in recruiting patients is an important issue in clinical trials [58-60], general practitioners may also experience problems in recruiting patients owing to their overload schedule, and our sample size is slightly lower than the expected. Finally, treatment directed to depression problems with the general practitioners in the iTAU group was not recorded. It would be necessary to consider these variables in the future to analyze possible influences on between-group results.

Our study has a significant strength: to the best of our knowledge, this is the first trial in Spain aimed at improving the symptomatology and quality of life of patients with depression using low-intensity interventions applied by the information and communication technologies. The treatment programs used in this study include therapeutic strategies based on mindfulness, healthy lifestyle, and positive affect, which have proven their efficacy for the treatment of depression; nevertheless, it is still the first study that adapts these interventions to information and communication technologies.

### Conclusions

This study has 3 important conclusions. First, 2 low-intensity, internet-based psychological interventions (HLP and MP) for the treatment of depression in primary care were more effective than iTAU at posttreatment. Second, all low-intensity, internet-based psychological interventions were also effective in improving medium- and long-term quality of life. Finally, PAPP was effective for improving health-related quality of life and well-being in patients with depression. Nevertheless, it is important to examine possible reasons that could be implicated in the ineffectiveness of PAPP in reducing depressive symptomatology, such as the intervention length, population, and treatment adherence, to increase its effectiveness in future studies of internet-based interventions programs for depression. Overall, our results suggest that although low-intensity, internet-based psychological programs are an efficacious therapeutic option for the treatment of depression in primary care, subsequent and more complex analyses are necessary to explain the reasons why some interventions appeared to affect some outcomes but not others. Furthermore, more research is still needed to assess the cost-effectiveness analysis of these interventions.

## Appendix

Multimedia Appendix 1 Primary outcome analysis with imputed data adjusted to Sex and Age (N=221): intervention comparisons along the follow-up.

Multimedia Appendix 2 Dose-response in imputed and adjusted (Sex and Age) primary outcome at post-treatment and along the follow-up.

Multimedia Appendix 3 SF-12 (Mental and Physical) analysis with imputed data adjusted to Sex and Age (N=221): intervention comparisons along the follow-up.

Multimedia Appendix 4 EuroQoL (VAS) and PHI analysis with imputed data adjusted to Sex and Age (N=221): intervention comparisons along the follow-up.

Multimedia Appendix 5 PANAS (positive and negative affect scales) analysis with imputed data adjusted to Sex and Age (N=221): intervention comparisons along the follow-up.

Multimedia Appendix 6 CONSORT-eHEALTH checklist (V 1.6.1).

## Abbreviations

CACE: Complier Average Causal Effect
CBT: cognitive behavioral therapy
HLP: healthy lifestyle program
iTAU: improved treatment as usual
MICE: Multiple Imputation with Chained Equation
MP: mindfulness program
NMW: national minimum wage
PANAS: Positive and Negative Affect Schedule
PAPP: positive affect promotion program
PHI: Pemberton Happiness Index
PHQ-9: Patient Health Questionnaire-9
SF-12: Short-Form Health Survey
VAS: visual analog scale
