# Supplementary material for: Efficacy of Three Low-Intensity, Internet-Based Psychological Interventions for the Treatment of Depression in Primary Care: Randomized Controlled Trial
Source: J Med Internet Res. 2020 Jun 5;22(6):e15845. doi: 10.2196/15845 (PMC7305559; doi:10.2196/15845)
Supplement: Multimedia Appendix 1 [file jmir_v22i6e15845_app1.docx]

**Multimedia Appendix 1.** Primary outcome analysis with imputed data adjusted to Sex and Age (N=221): intervention comparisons along the follow-up^a^

| **PHQ-9** | | | | **Time 1** | | **Time 2** | | **Time 3** | | **Time 4** | |
| --- | --- | --- | --- | --- | --- | --- | --- | --- | --- | --- | --- |
|  |  |  |  | (pre-treatment) | | (post-treatment) | | (6 months) | | (12 months) | |
| **iTAU  vs HLP** | *P* | | | .20 | | .01 | | .26 | | .75 | |
|  | B (95% CI) | | | 1.41 (-0.75 to 3.58) | | -3.05 (-5.42 to -0.66) | | -1.41 (-3.87 to 1.05) | | -0.37 (-2.70 to 1.95) | |
| **iTAU vs MP** | *P* | | | .42 | | .01 | | .15 | | .97 | |
|  | B (95% CI) | | | 0.89 (-1.29 to 3.08) | | -3.19 (-5.59 to -0.80) | | -1.83 (-4.31 to 0.65) | | 0.04 (-2.30 to 2.38) | |
| **iTAU  vs PAPP** | *P* | | | .21 | | .22 | | .11 | | >.99 | |
|  | B (95% CI) | | | 1.36 (-0.79 to 3.52) | | -1.50 (-3.86 to 0.86) | | -1.99 (-4.43 to 0.45) | | 0.00 (-2.30 to 2.31) | |
| **HLP  vs MP** | *P* | | | .65 | | .90 | | .74 | | .73 | |
|  | B (95% CI) | | | 0.51 (-1.71 to 2.73) | | 0.17 (-2.33 to 2.67) | | -0.46 (-2.81 to 1.88) | | -0.41 (-2.79 to 1.96) | |
| **HLP  vs PAPP** | *P* | | | .97 | | .21 | | .64 | | .75 | |
|  | B (95% CI) | | | 0.04 (-2.14 to 2.22) | | -1.54 (-3.93 to 0.85) | | 0.58 (-1.89 to 3.05) | | -0.38 (-2.71 to 1.96) | |
| **MP  vs PAPP** | *P* | | | .67 | | .17 | | .90 | | .98 | |
|  | B (95% CI) | | | 0.47 (-1.72 to 2.66) | | 1.69 (-0.71 to 4.09) | | -0.16 (-2.64 to 2.32) | | -0.03 (-2.38 to 2.31) | |
|  | | |  |  | |  | |  | |  | |

^a^g: Hedge’s effect size measure; *P*: P value; statistically significant values (*P*<.05) are shown in italics; B: regression coefficients; 95% CI: Confidence interval at 95%.
