# Supplementary material for: Efficacy of Three Low-Intensity, Internet-Based Psychological Interventions for the Treatment of Depression in Primary Care: Randomized Controlled Trial
Source: J Med Internet Res. 2020 Jun 5;22(6):e15845. doi: 10.2196/15845 (PMC7305559; doi:10.2196/15845)
Supplement: Multimedia Appendix 2 [file jmir_v22i6e15845_app2.docx]

**Multimedia Appendix 2.** Dose-response in imputed and adjusted (Sex and Age) primary outcome at post-treatment and along the follow-up

|  |  | **Pre-treatment to  Post-treatment** | |  | **Pre-treatment to**  **Post-treatment** | |  | **Pre-treatment to  Post-treatment** | |
| --- | --- | --- | --- | --- | --- | --- | --- | --- | --- |
|  |  | B^a^ (95% CI) | *P* |  | B (95% CI) | *P* |  | B (95% CI) | *P* |
| **HLP** |  |  |  |  |  |  |  |  |  |
| CACE analysis^b^ |  | -3.15 (4.29 to -2.02) | *.008* |  | 0.23 (1.28 to 1.73) | .89 |  | -0.54 (2.16 to 1.09) | .74 |
| Effect per session |  | -0.28 (0.35 to -0.21) | *.001* |  | -0.17 (0.25 to -0.09) | .*03* |  | -0.06 (0.13 to 0.02) | .46 |
| **MP** |  |  |  |  |  |  |  |  |  |
| CACE analysis |  | 1.10 (0.59 to 2.79) | .52 |  | 0.80 (0.97 to 2.58) | .65 |  | 1.49 (0.18 to 3.15) | .38 |
| Effect per session |  | -0.28 (0.35 to -0.21) | *.001* |  | -0.17 (0.25 to -0.09) | .*03* |  | -0.06 (0.13 to 0.02) | .46 |
| **PAPP** |  |  |  |  |  |  |  |  |  |
| CACE analysis |  | -0.25 (1.90 to 1.40) | .89 |  | 0.08 (1.61 to 1.78) | .96 |  | -0.84 (2.42 to 0.74) | .60 |
| Effect per session |  | -0.28 (0.35 to -0.21) | .*001* |  | -0.17 (0.25 to -0.09) | *.03* |  | -0.06 (0.13 to 0.02) | .46 |
| **All** |  |  |  |  |  |  |  |  |  |
| CACE analysis |  | -2.14 (2.90 to -1.39) | *.005* |  | -0.60 (1.47 to 0.28) | .50 |  | -0.25 (1.07 to 0.57) | .76 |
| Effect per session |  | -0.28 (0.35 to -0.21) | *.001* |  | -0.17 (0.25 to -0.09) | .*03* |  | -0.06 (0.13 to 0.02) | .46 |

^a^B: regression coefficients; 95% CI: Confidence interval at 95; *P*: P value; statistically significant values (*P*<.05) are shown in italics.
^b^Compliance as attendance > 4 modules.
