# Supplementary material for: Efficacy of Three Low-Intensity, Internet-Based Psychological Interventions for the Treatment of Depression in Primary Care: Randomized Controlled Trial
Source: J Med Internet Res. 2020 Jun 5;22(6):e15845. doi: 10.2196/15845 (PMC7305559; doi:10.2196/15845)
Supplement: Multimedia Appendix 3 [file jmir_v22i6e15845_app3.docx]

**Multimedia Appendix 3.** SF-12 (Mental and Physical) analysis with imputed data adjusted to Sex and Age (N=221): intervention comparisons along the follow-up^a^

| **Mental Scale SF-12** | | **Time 1** | **Time 2** | **Time 3** | **Time 4** |
| --- | --- | --- | --- | --- | --- |
|  |  | (pre-treatment) | (post-treatment) | (6 months) | (12 months) |
| **iTAU  vs HLP** | *P* | .83 | .03 | .57 | .99 |
|  | B (95% CI) | -0.38 (-3.88 to 3.12) | -4.93 (-9.40 to -0.45) | -1.39 (-6.16 to 3.39) | 0.02 (-4.99 to 5.03) |
| **iTAU vs MP** | *P* | .26 | .26 | .91 | .82 |
|  | B (95% CI) | -2.04 (-5.57 to 1.49) | -2.58 (-7.09 to 1.93) | -0.27 (-5.08 to 4.55) | 0.58 (-4.46 to 5.63) |
| **iTAU  vs PAPP** | *P* | .48 | *.001* | .31 | .22 |
|  | B (95% CI) | -1.26 (-4.73 to 2.22) | -7.4 (-11.84 to -2.96) | -2.46 (-7.2 to 2.28) | -3.12 (-8.09 to 1.85) |
| **HLP  vs MP** | *P* | .36 | .31 | .65 | .83 |
|  | B (95% CI) | -1.66 (-5.24 to 1.92) | 1.35 (-3.48 to 6.18) | 0.68 (-4.37 to 5.73) | 0.56 (-4.55 to 5.68) |
| **HLP  vs PAPP** | *P* | .99 | .36 | .74 | .46 |
|  | B (95% CI) | 0.03 (-4.04 to 4.1) | -2.05 (-6.47 to 2.37) | 0.7 (-3.41 to 4.81) | 1.39 (-2.33 to 5.12) |
| **MP  vs PAPP** | *P* | .10 | .13 | .17 | .37 |
|  | B (95% CI) | -3.41 (-7.5 to 0.69) | -3.47 (-7.91 to 0.97) | -2.88 (-7.01 to 1.25) | -1.70 (-5.45 to 2.05) |

| **Physical Scale SF-12** | | **Time 1** | **Time 2** | **Time 3** | **Time 4** |
| --- | --- | --- | --- | --- | --- |
|  |  | (pre-treatment) | (post-treatment) | (6 months) | (12 months) |
| **iTAU  vs HLP** | *P* | .96 | .07 | .04 | .23 |
|  | B (95% CI) | 0.10 (-3.95 to 4.16) | 4.14 (-0.26 to 8.55) | 4.42 (0.32 to 8.51) | -2.25 (-5.96 to 1.47) |
| **iTAU vs MP** | *P* | .09 | .01 | *.001* | .66 |
|  | B (95% CI) | 3.54 (-0.55 to 7.62) | 5.56 (1.13 to 10) | 8.00 (3.88 to 12.13) | 0.85 (-2.89 to 4.59) |
| **iTAU  vs PAPP** | *P* | .95 | .35 | .01 | .65 |
|  | B (95% CI) | 0.13 (-3.89 to 4.16) | 2.10 (-2.27 to 6.46) | 5.12 (1.06 to 9.18) | -0.85 (-4.54 to 2.83) |
| **HLP  vs MP** | *P* | .10 | .54 | .09 | .11 |
|  | B (95% CI) | 3.44 (-0.71 to 7.58) | 3.28 (-0.92 to 7.49) | 2.34 (-1.57 to 6.25) | 3.10 (-0.70 to 6.89) |
| **HLP  vs PAPP** | *P* | .62 | .28 | .66 | .22 |
|  | B (95% CI) | -0.88 (-4.39 to 2.64) | -2.48 (-6.97 to 2.02) | -1.08 (-5.87 to 3.72) | -3.14 (-8.17 to 1.88) |
| **MP  vs PAPP** | *P* | .66 | .04 | .37 | .15 |
|  | B (95% CI) | 0.78 (-2.75 to 4.32) | -4.83 (-9.34 to -0.31) | -2.19 (-7.01 to 2.63) | -3.71 (-8.76 to 1.35) |

^a^g: Hedge’s effect size measure; *P*: P value; statistically significant values (*P*<.05) are shown in italics; B: regression coefficients; 95% CI: Confidence interval at 95%.
