# Supplementary material for: Efficacy of Three Low-Intensity, Internet-Based Psychological Interventions for the Treatment of Depression in Primary Care: Randomized Controlled Trial
Source: J Med Internet Res. 2020 Jun 5;22(6):e15845. doi: 10.2196/15845 (PMC7305559; doi:10.2196/15845)
Supplement: Multimedia Appendix 4 [file jmir_v22i6e15845_app4.docx]

**Multimedia Appendix 4.** EuroQoL (VAS) and PHI analysis with imputed data adjusted to Sex and Age (N=221): intervention comparisons along the follow-up^a^

| **EuroQoL**  **(VAS)** | | | | **Time 1** | **Time 3** | **Time 4** | |
| --- | --- | --- | --- | --- | --- | --- | --- |
|  |  |  |  | (pre-treatment) | (6 months) | (12 months) | |
| **iTAU  vs HLP** | | *P* | | .87 | .31 | .07 | |
|  |  | B (95% CI) | | 0.71 (-7.66 to 9.07) | -3.83 (-11.23 to 3.56) | -6.87 (-14.17 to 0.43) | |
| **iTAU vs MP** | | *P* | | .24 | .33 | .44 | |
|  |  | B (95% CI) | | -5.08 (-13.52 to 3.35) | -3.71 (-11.16 to 3.74) | -2.86 (-10.21 to 4.49) | |
| **iTAU  vs PAPP** | | *P* | | .36 | .59 | .58 | |
|  |  | B (95% CI) | | -3.87 (-12.18 to 4.43) | -1.99 (-9.33 to 5.34) | -2.04 (-9.29 to 5.20) | |
| **HLP  vs MP** | | *P* | | .18 | .97 | .37 | |
|  |  | B (95% CI) | | -5.79 (-14.34 to 2.76) | -2.04 (-9.29 to 5.2) | 3.39 (-4.02 to 10.79) | |
| **HLP  vs PAPP** | | *P* | | .28 | .63 | .20 | |
|  |  | B (95% CI) | | -4.58 (-12.98 to 3.82) | 1.84 (-5.58 to 9.26) | 4.82 (-2.5 to 12.15) | |
| **MP  vs PAPP** | | *P* | | .78 | .65 | .83 | |
|  |  | B (95% CI) | | 1.21 (-7.23 to 9.65) | 1.71 (-5.74 to 9.17) | 0.82 (-6.54 to 8.18) | |
|  | | | |  |  |  | |
| **PHI** | | | | **Time 1** | **Time 3** | **Time 4** | |
|  |  |  |  | (pre-treatment) | (6 months) | (12 months) | |
| **iTAU  vs HLP** | *P* | | | .87 | .30 | .35 | |
|  | B (95% CI) | | | -0.06 (-0.76 to 0.64) | 0.40 (-0.36 to 1.16) | -0.32 (-0.98 to 0.34) | |
| **iTAU vs MP** | *P* | | | .59 | .01 | .81 | |
|  | B (95% CI) | | | -0.19 (-0.9 to 0.51) | 1.01 (0.24 to 1.77) | 0.08 (-0.58 to 0.75) | |
| **iTAU  vs PAPP** | *P* | | | .63 | .03 | .62 | |
|  | B (95% CI) | | | -0.17 (-0.87 to 0.52) | 0.82 (0.07 to 1.58) | -0.17 (-0.82 to 0.49) | |
| **HLP  vs MP** | *P* | | | .71 | .12 | .25 | |
|  | B (95% CI) | | | -0.13 (-0.85 to 0.58) | 0.43 (-0.23 to 1.1) | 0.40 (-0.28 to 1.07) | |
| **HLP  vs PAPP** | *P* | | | .76 | .27 | .66 | |
|  | B (95% CI) | | | -0.11 (-0.81 to 0.59) | 0.42 (-0.34 to 1.19) | 0.15 (-0.51 to 0.81) | |
| **MP  vs PAPP** | *P* | | | .95 | .63 | .46 | |
|  | B (95% CI) | | | 0.02 (-0.68 to 0.73) | -0.19 (-0.95 to 0.58) | -0.25 (-0.92 to 0.42) | |
|  | | |  |  |  |  |  |

^a^g: Hedge’s effect size measure; *P*: P value; statistically significant values (*P*<.05) are shown in italics; B: regression coefficients; 95% CI: Confidence interval at 95%.
