# Supplementary material for: Efficacy of Three Low-Intensity, Internet-Based Psychological Interventions for the Treatment of Depression in Primary Care: Randomized Controlled Trial
Source: J Med Internet Res. 2020 Jun 5;22(6):e15845. doi: 10.2196/15845 (PMC7305559; doi:10.2196/15845)
Supplement: Multimedia Appendix 5 [file jmir_v22i6e15845_app5.docx]

**Multimedia Appendix 5.** PANAS (positive and negative affect scales) analysis with imputed data adjusted to Sex and Age (N=221): intervention comparisons along the follow-up^a^

| **Positive Affect PANAS** | | | **Time 1** | | **Time 2** | **Time 3** | | **Time 4** | |
| --- | --- | --- | --- | --- | --- | --- | --- | --- | --- |
|  |  |  | (pre-treatment) | | (post-treatment) | (6 months) | | (12 months) | |
| **iTAU  vs HLP** | *P* | | .69 | | .95 | .16 | | .07 | |
|  | B (95% CI) | | 0.50 (-1.92 to 2.91) | | 0.10 (-3.21 to 3.42) | 2.33 (-0.92 to 5.58) | | 3.44 (-0.23 to 7.11) | |
| **iTAU vs MP** | *P* | | .44 | | .91 | .42 | | .10 | |
|  | B (95% CI) | | -0.96 (-3.40 to 1.47) | | -0.18 (-3.52 to 3.15) | 1.35 (-1.93 to 4.62) | | 3.13 (-0.57 to 6.83) | |
| **iTAU  vs PAPP** | *P* | | .27 | | .13 | .86 | | .40 | |
|  | B (95% CI) | | -1.36 (-3.76 to 1.04) | | -2.56 (-5.85 to 0.72) | -0.30 (-3.52 to 2.93) | | 1.59 (-2.06 to 5.23) | |
| **HLP  vs MP** | *P* | | .25 | | .87 | .56 | | .87 | |
|  | B (95% CI) | | -1.46 (-3.93 to 1.01) | | -0.89 (-4.17 to 2.39) | -0.17 (-3.87 to 3.54) | | -0.31 (-4.06 to 3.44) | |
| **HLP  vs PAPP** | *P* | | .13 | | .12 | .11 | | .32 | |
|  | B (95% CI) | | -1.85 (-4.28 to 0.57) | | -2.67 (-5.99 to 0.66) | -2.63 (-5.89 to 0.63) | | -1.85 (-5.54 to 1.83) | |
| **MP  vs PAPP** | *P* | | .75 | | .16 | .32 | | .41 | |
|  | B (95% CI) | | -0.40 (-2.83 to 2.04) | | -2.38 (-5.72 to 0.96) | -1.65 (-4.92 to 1.63) | | -1.55 (-5.25 to 2.16) | |
|  | | |  | |  |  | |  | |
| **Negative Affect PANAS** | | | **Time 1** | | **Time 2** | **Time 3** | | **Time 4** | |
|  |  |  | (pre-treatment) | | (post-treatment) | (6 months) | | (12 months) | |
| **iTAU  vs HLP** | *P* | | .49 | | .95 | .42 | | .08 | |
|  | B (95% CI) | | -1.12 (-4.29 to 2.05) | | 0.10 (-3.21 to 3.42) | -1.29 (-4.45 to 1.86) | | -2.98 (-6.32 to 0.36) | |
| **iTAU vs MP** | *P* | | .75 | | .91 | .43 | | .15 | |
|  | B (95% CI) | | 0.52 (-2.68 to 3.71) | | -0.18 (-3.52 to 3.15) | -1.28 (-4.46 to 1.90) | | -2.50 (-5.86 to 0.87) | |
| **iTAU  vs PAPP** | *P* | | .17 | | .13 | .13 | | .04 | |
|  | B (95% CI) | | -2.22 (-5.36 to 0.93) | | -2.56 (-5.85 to 0.72) | -2.44 (-5.57 to 0.7) | | -3.49 (-6.80 to -0.17) | |
| **HLP  vs MP** | *P* | | .32 | | .87 | .99 | | .78 | |
|  | B (95% CI) | | 1.64 (-1.60 to 4.88) | | 0.02 (-3.17 to 3.20) | 0.83 (-2.57 to 4.23) | | 0.48 (-2.90 to 3.90) | |
| **HLP  vs PAPP** | *P* | | .50 | | .12 | .49 | | .77 | |
|  | B (95% CI) | | -1.09 (-4.28 to 2.09) | | -2.67 (-5.99 to 0.66) | -1.14 (-4.31 to 2.03) | | -0.5 (-3.86 to 2.85) | |
| **MP  vs PAPP** | *P* | | .09 | | .16 | .47 | | .56 | |
|  | B (95% CI) | | -2.73 (-5.93 to 0.46) | | -2.38 (-5.72 to 0.96) | -1.16 (-4.34 to 2.02) | | -0.99 (-4.36 to 2.38) | |
|  | | |  |  |  | |  |  | |

^a^g: Hedge’s effect size measure; *P*: P value; statistically significant values (*P*<.05) are shown in italics; B: regression coefficients; 95% CI: Confidence interval at 95%.
